# Supplementary material for: Regulatory T cells use heparanase to access IL-2 bound to extracellular matrix in inflamed tissue
Source: Nat Commun. 2024 Feb 20;15:1564. doi: 10.1038/s41467-024-45012-9 (PMC10879116; doi:10.1038/s41467-024-45012-9)
Supplement: Supplementary file 1 — Supplementary Information [file 41467_2024_45012_MOESM1_ESM.pdf]

Supplementary Information for

Regulatory T cells use heparanase to access IL-2 bound to extracellular matrix in inflamed tissues

Hunter A. Martinez, Ievgen Koliesnik, Gernot Kaber, Jacqueline K. Reid, Nadine Nagy, Graham Barlow, Ben A. Falk, Carlos O. Medina, Aviv Hargil, Svenja Zihsler, Israel Vlodavsky, Jin-Ping Li, Magdiel Pérez-Cruz, Sai-Wen Tang, Everett H. Meyer, Lucile E. Wrenshall, James D. Lord, K. Christopher Garcia, Theo D. Palmer, Lawrence Steinman, Gerald T. Nepom, Thomas N. Wight, Paul L. Bollyky, and Hedwich F. Kuipers

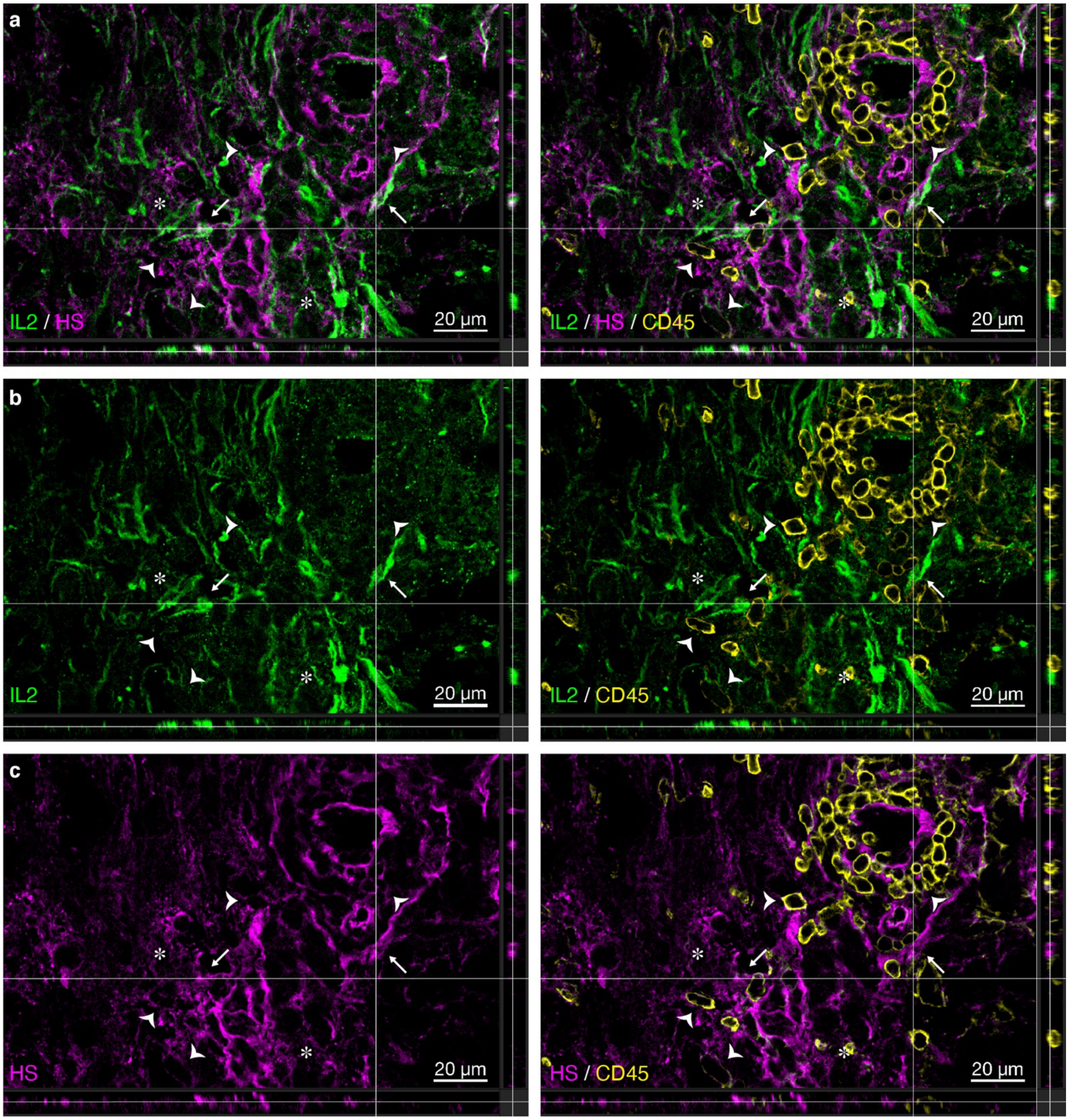

Supplementary Figure 1. HS and IL2 colocalize in inflamed CNS tissue. Orthogonal views of z-stacks of EAE spinal cord tissue (29 dpi) stained for IL-2 (green), HS (magenta) and CD45 (yellow). Shown are the overlay of IL-2 and HS staining (**a**, overlay with appear white/grey), as well as individual IL-2 (**b**) and HS (**c**) channels. Arrows indicate overlap of IL-2 and HS staining on cellular structures, arrowheads indicate HS staining associated with CD45 cells and asterisks indicate diffuse IL-2 and HS staining.

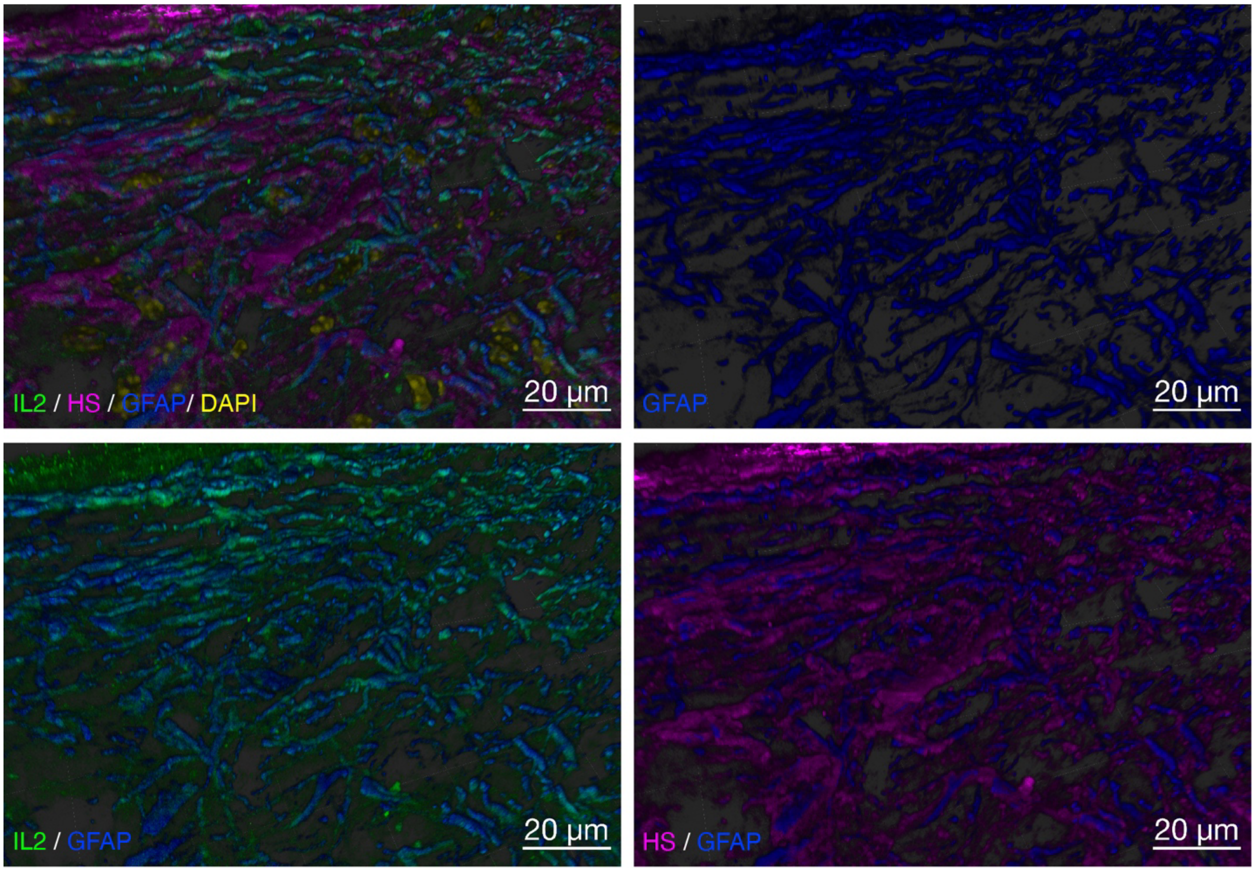

Supplementary Figure 2. Immunohistochemical analysis of GFAP, HS and IL2 in EAE CNS tissue. Imaris 3D surface rendering of EAE spinal cord tissue stained for GFAP (blue), IL-2 (green) and HS (magenta), showing association of both IL-2 and HS staining with GFAP positive structures. DAPI counterstain for nuclei is shown in yellow.

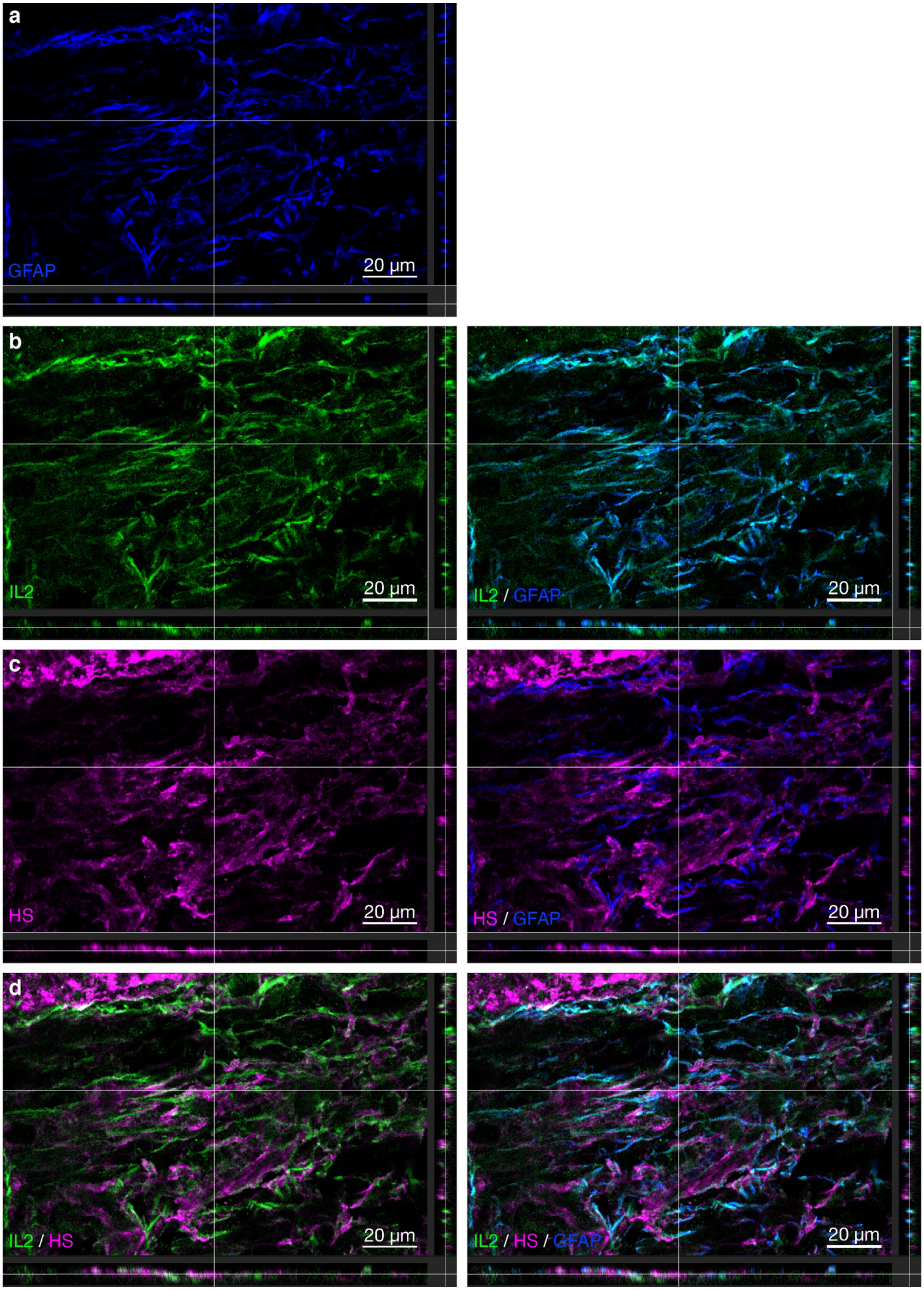
a
GFAP
20 µm
b
IL2
20 µm
IL2 / GFAP
20 µm
c
HS
20 µm
HS / GFAP
20 µm
d
IL2 / HS
20 µm
IL2 / HS / GFAP
20 µm

Supplementary Figure 3. IL-2 and HS are associated with reactive astrocytes. Orthogonal views of z-stacks of EAE spinal cord tissue stained for GFAP (blue), IL-2 (green) and HS (magenta). Shown are individual **a,** GFAP, **b,** IL-2 and **c,** HS channels, as well as **d,** the overlay of IL-2 and HS staining (overlay will appear white/grey), alone and in conjunction with GFAP staining.

a

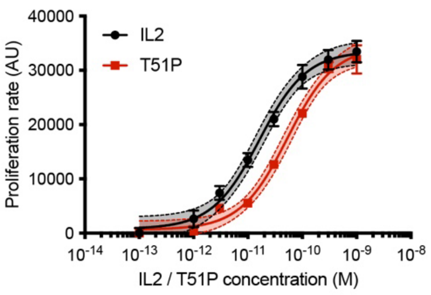

	EC50 (M)	95% CI (M)	R^2 (non -linear regression)
IL2	1.622e-011	1.077e-011 to 2.443e-011	0.9803
T51P	5.353e-011	3.775e-011 to 7.589e-011	0.9846

b

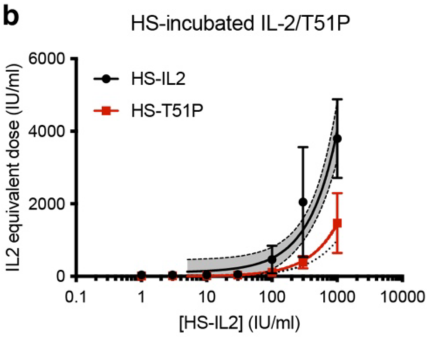

HS-bound concentration (IU/ml)	Equivalent soluble concentration (IU/ml) [fold increase]	
	IL2	T51P
1	113 [113 x]	- * [-]
3	120 [40 x]	- * [-]
10	148 [14.8 x]	6 [0.6 x]
30	226 [7.53 x]	35 [1.1 x]
100	500 [5 x]	138 [1.38x]
300	1281 [4.27 x]	430 [1.43 x]
1000	4018 [4.018 x]	1452 [1.452 x]
R^2 (linear regression)	0.9486	0.9979

c

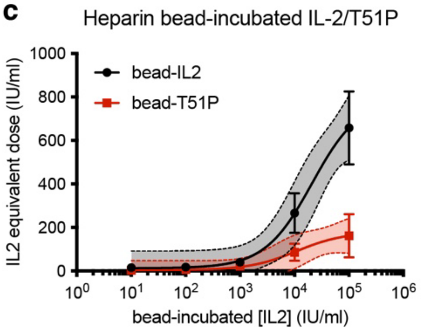

Bead-incubated concentration (IU/ml)	Equivalent soluble concentration (IU/ml)	
	IL2	T51P
$1*10^1$	16	3
$1*10^2$	17	6
$1*10^3$	41	23
$1*10^4$	266	86
$1*10^5$	658	162
R^2 (non-linear regression)	0.821	0.6482

Supplementary Figure 4. Quantification of effective doses of HS-bound and heparin-coated bead-bound IL-2 and T51P-IL-2. Shown are mean +/- SEM for experimental values, best-fit curves for each analysis and 95% confidence areas (shaded areas). **a,** Non-linear regression analysis (left; variable slope, 3 parameters) and calculated EC50 values (right) of proliferation of CTLL2 cells induced by soluble IL-2 and T51P-IL-2. **b,** Linear regression analysis (left) and extrapolated effective doses of HS-bound IL-2 and T51P-IL-2 assessed by induction of proliferation of CTLL2 cells (n=2-4 experiments). The dotted line depicts a theoretical curve of equal effectiveness (comparing bound IL-2 to soluble IL-2). Table (right) shows extrapolated equivalent doses delivered by HS-bound IL-2 concentrations, depicting which dose of soluble IL-2, or T51P-IL-2, elicits a similar proliferative response as the dose of HS-bound IL-2, or T51P-IL-2, shown in the left column. Fold increase of proliferation rate over the rate induced by the same unbound cytokine is indicated as well. * below detection. **c,** Non-linear regression analysis (left; variable slope, 4 parameters) and extrapolated effective doses of heparin-coated bead-bound IL-2 and T51P-IL-2, assessed by induction of proliferation of CTLL2 cells (n=3 experiments). Table (right) shows extrapolated equivalent doses IL-2 delivered by heparin coated

beads pre-incubated with various concentrations of IL-2, depicting which concentration of soluble IL-2, or T51-P, elicits a similar proliferative response as heparin beads pre-incubated with the concentration of IL-2, or T51P-IL-2, shown in the left column.

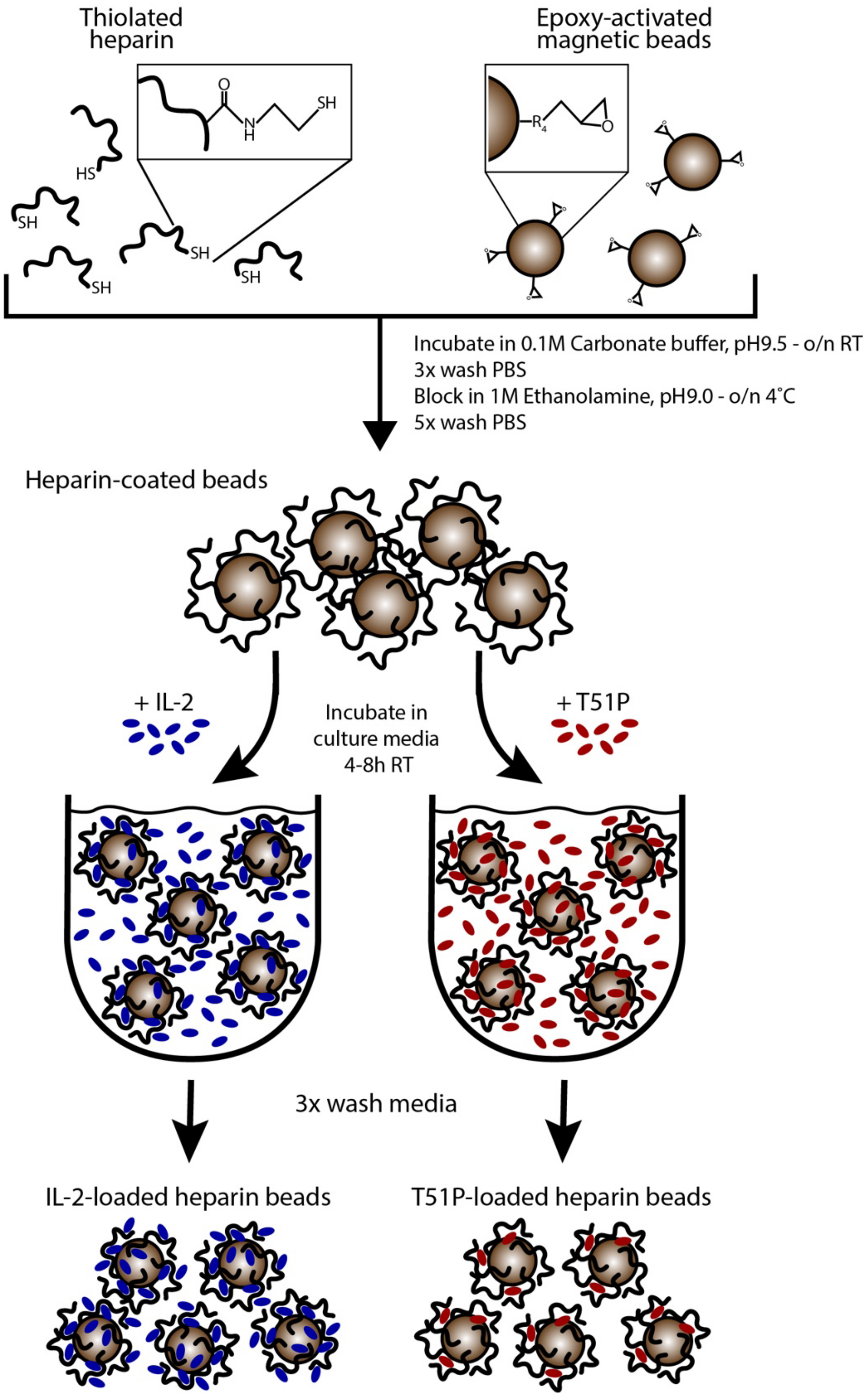

Supplementary Figure 5. Generation of IL-2-loaded heparin-coated beads. Schematic overview of generation of IL-2-loaded, heparin-coated magnetic beads. Thiolated heparin is coupled to 1 μm epoxy-activated magnetic beads to yield heparin-coated beads. These beads are then incubated with IL-2 or T51P-IL-2 in cell culture media (RPMI containing 10% FCS and penicillin/streptomycin). After washing, these IL-2 or T51P-loaded beads are used to deliver IL-2 to cells in culture.

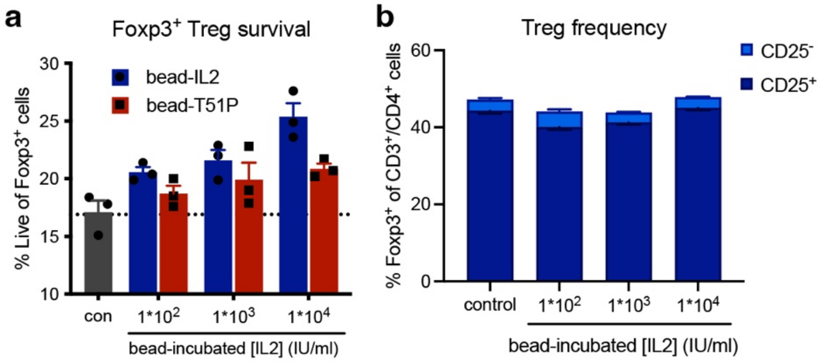

Supplementary Figure 6. HS-bound IL-2 supports the viability, but not induction of Treg. **a**, Absolute frequencies of live cells among FOXP3$^+$ CD4+ cell cultures with IL-2 loaded beads. Viability of FOXP3$^+$ Treg among CD4$^+$ T cells cultured in the presence of heparin-coated beads pre-incubated with IL-2 or T51P-IL-2 (T51P). Cells were analyzed by flow cytometry 24 hr after start of culture. Absolute percentages of viable cells among FOXP3$^+$ CD4+ gated cells are depicted. Background viability of cells incubated in media only (con; containing no IL-2) is indicated by a dotted line. **b,** Frequency of Foxp3+ cells among CD25+-enriched CD4+ T cells cultured in the presence of heparin-coated beads pre-incubated with different concentrations of IL-2, or media alone (control). Shown are the fractions of persisting Foxp3$^+$ cells retaining the PE-conjugated anti-CD25 antibody used for enrichment, as well as newly induced CD25$^-$ Foxp3$^+$ cells. **c,** Viability of CD25$^-$ FOXP3$^-$ Tconv among CD25+-enriched CD4+ T cells cultured in the presence of heparin-coated beads pre-incubated with IL-2 or T51P-IL-2 (T51P). *P* value depicts variation due to the cytokine that the beads were incubated with (IL-2 vs. T51P-IL-2), determined by two-way ANOVA

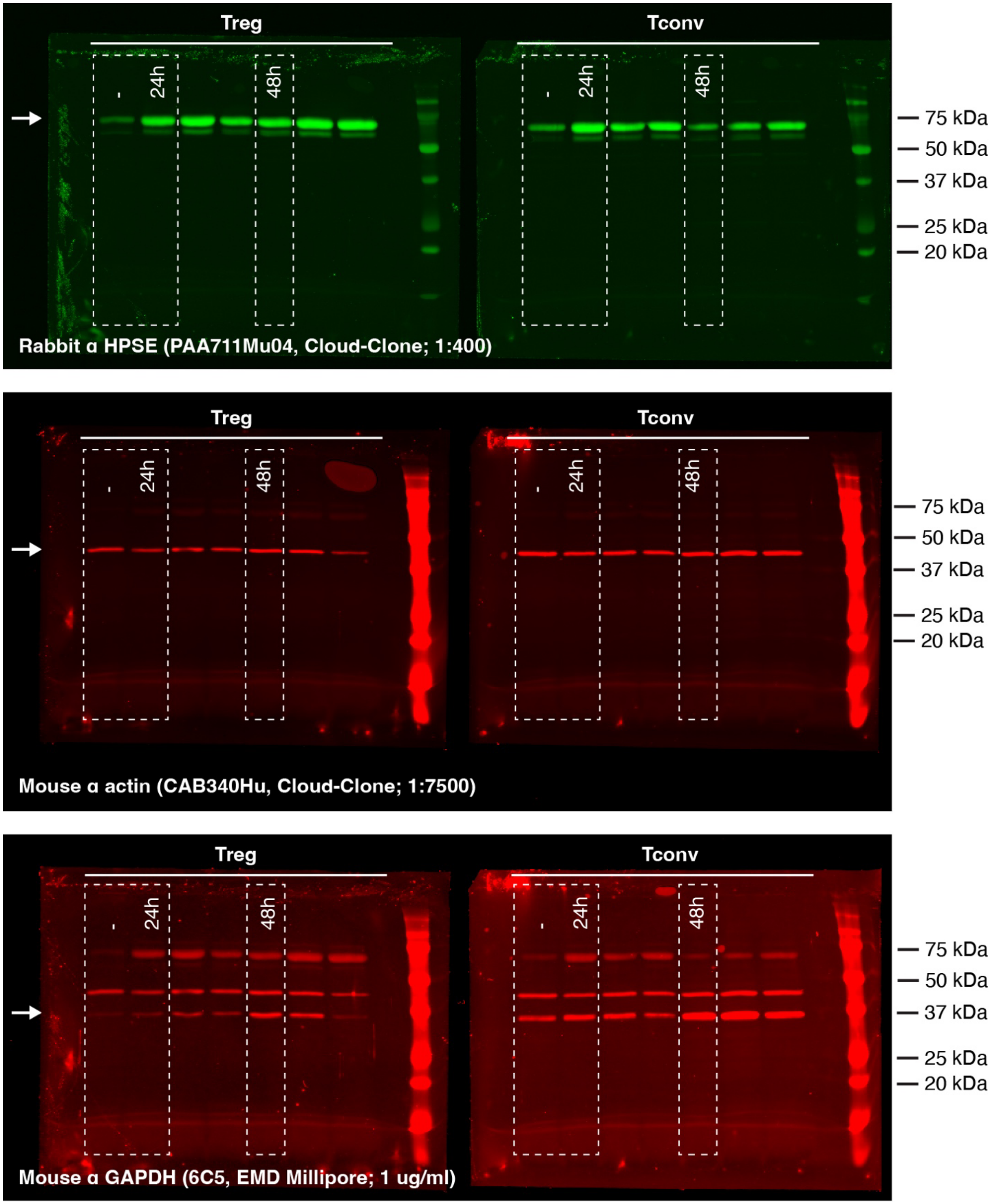

Supplementary Figure 7. Uncropped Western blots for analysis of HPSE expression in Treg and Tconv as shown in Figure 3b. Shown are the membranes stained for HPSE (top), actin (middle) and GAPDH (bottom). The samples shown in Figure 3b are outlined. Note that there is residual staining from the previous stains in the bottom panel.

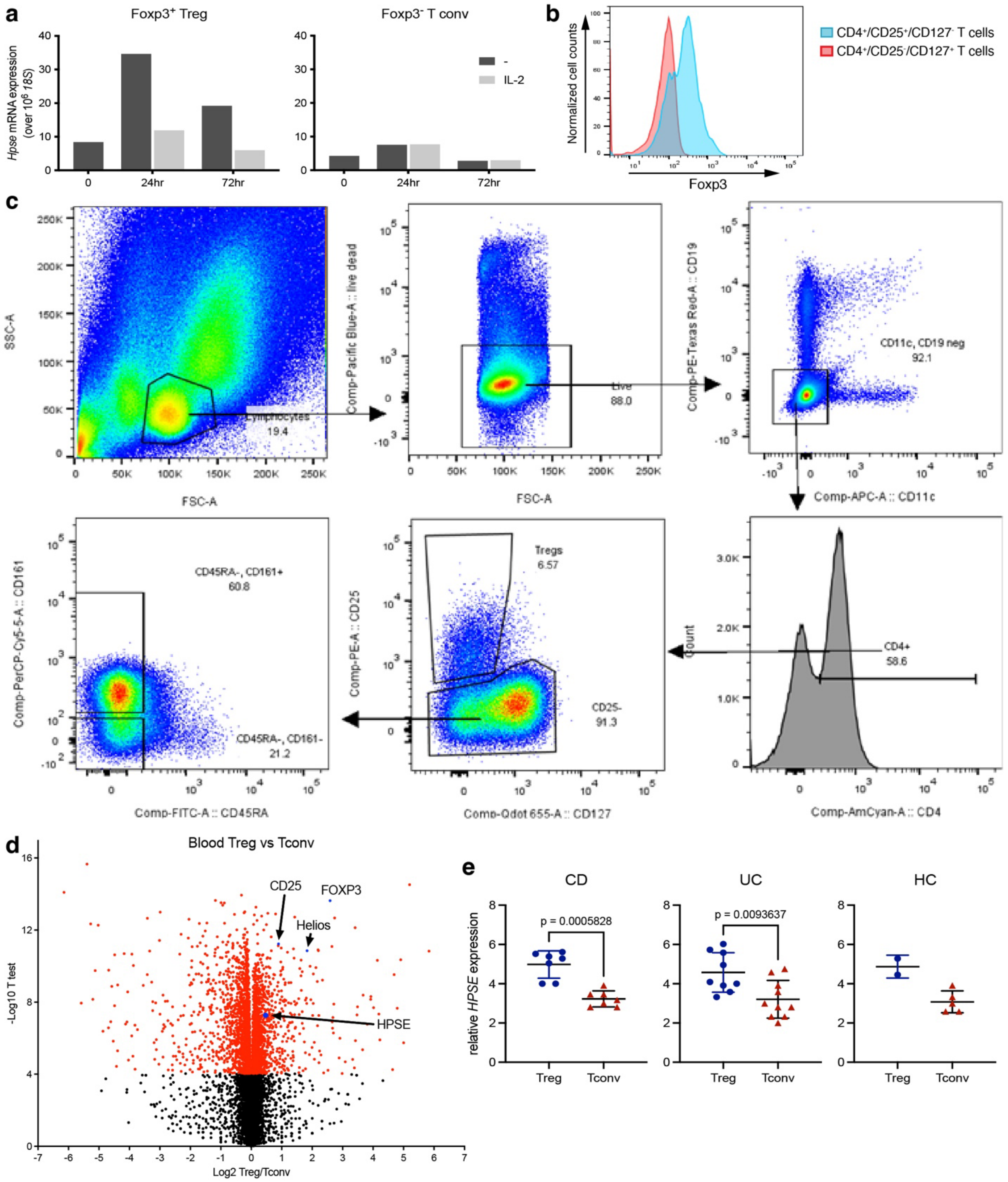

Supplementary Figure 8. Treg express higher levels of HPSE than Tconv. a, Upregulation of HPSE in Treg is regulated by IL-2: qPCR quantitation of *Hpse* mRNA expression in FACS sorted murine Foxp3^{+} Treg and Foxp3^{-} Tconv after *in vitro* activation with aCD3/aCD28, either alone, or in the presence of IL-2 (200 IU/ml). mRNA expression was normalized by *18S* mRNA expression. **b,** Confirmation of Foxp3 expression in FACS sorted human Treg**.** Foxp3 protein

expression in FACS sorted and *in vitro* activated human CD4+/CD25+/CD127- Treg (blue histogram) and CD4+/CD25-/CD127+ Tconv (red histogram). **c,** Human Treg express higher levels of HPSE than Tconv: Gating scheme showing the Treg and Teff populations that were isolated from human blood and colon samples and analyzed for gene expression. **d,** Volcano plots depicting relative expression of genes in Treg over Teff in human blood. **e,** Relative *HPSE* mRNA expression in FACS sorted human Treg and Tconv isolated from surgically resected colons from patients with Crohn's Disease (CD), Ulcerative Colitis (UC) and healthy controls (HC). Shown are mean relative HPSE expression +/- SD; P values determined by Mann-Whitney test.

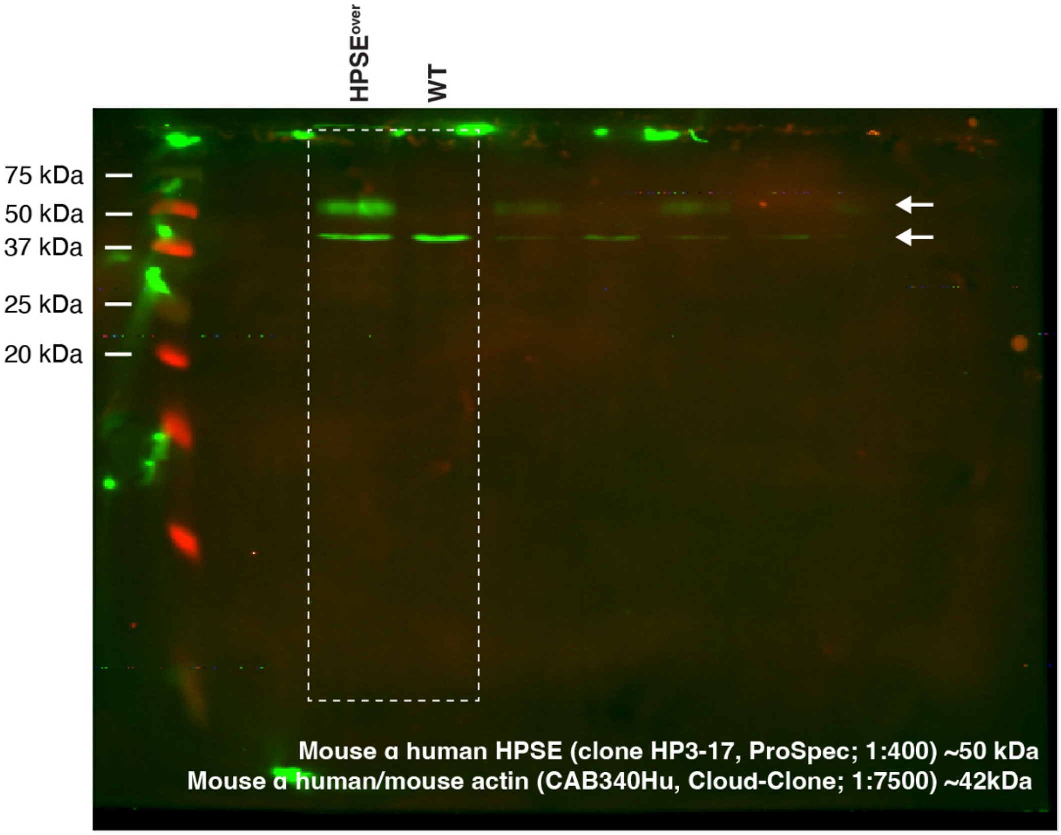

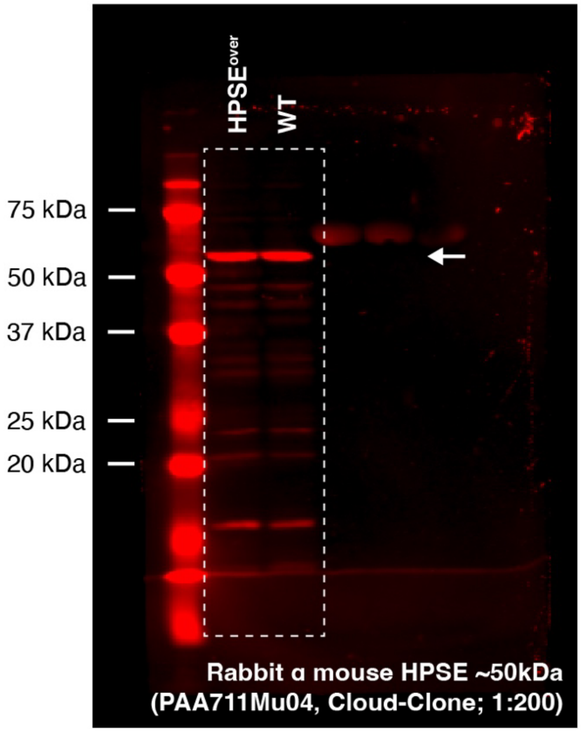

Supplementary Figure 9. Uncropped Western blots for analysis of HPSE expression in CTLL2 cells overexpressing human HPSE, or WT CTLL2, as shown in Figure 4a.

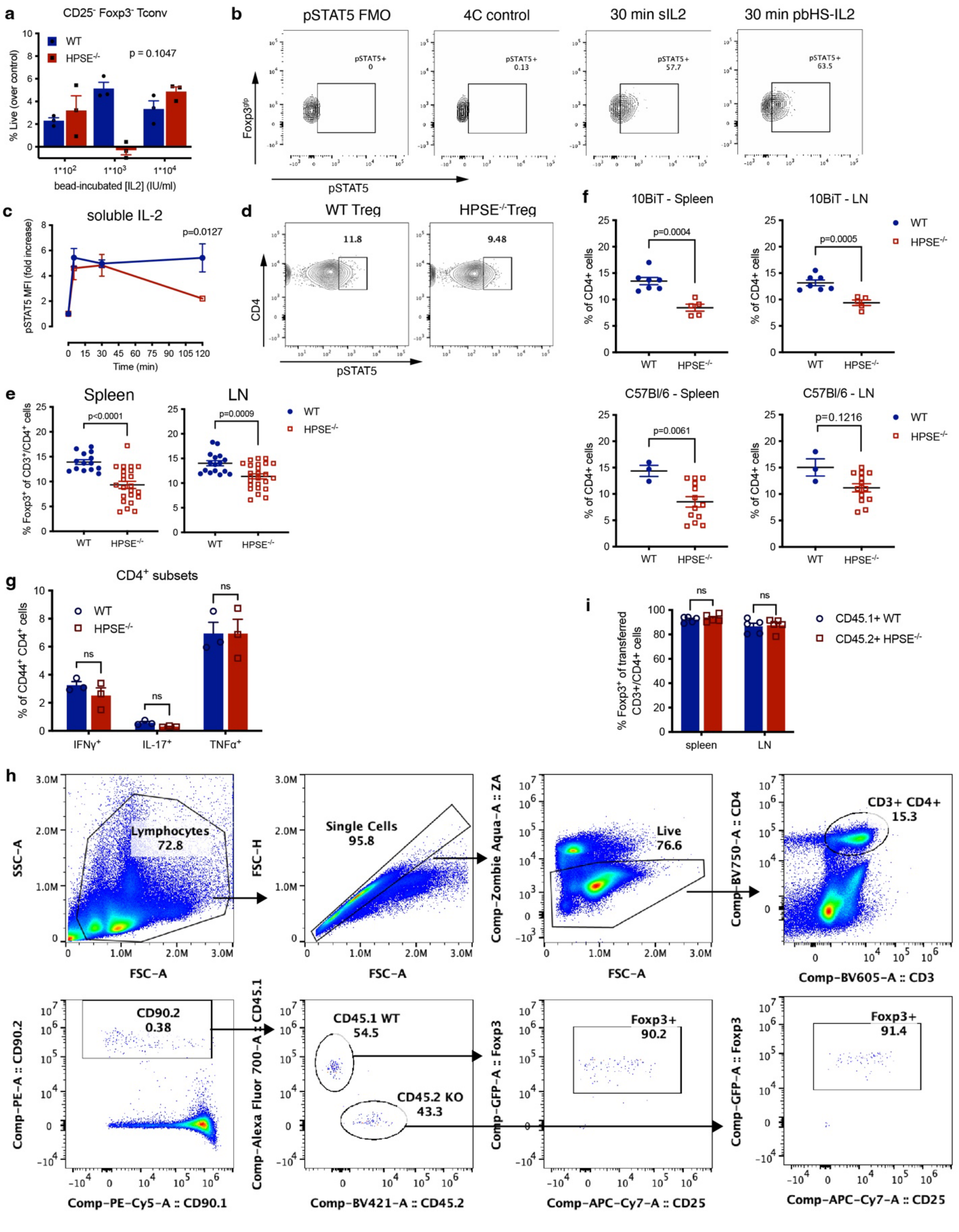

Supplementary Figure 10. HPSE expression supports Foxp3+ Treg access to IL-2 and their homeostasis *in vitro* and *in vivo*. a, Viability of WT and HPSE$^{-/-}$ CD25^{-} Foxp3^{-} Tconv among

$CD4^+$ T cells cultured in the presence of heparin-coated beads pre-incubated with increasing concentrations of IL-2. Viability was measured by flow cytometry 24 hr after start of culture and is shown as percent viable cells corrected for baseline viability of cell cultured in media alone. Shown is a representative of 4 independent experiments (mean + SEM of triplicate samples); *P* value depicts variation due to the genotype (WT vs. $HPSE^{-/-}$), determined by two-way ANOVA. **b,** Representative plots of phosphorylated Stat5 (pSTAT5) staining, assessed by flow cytometry, in unstimulated cells (kept at 4°C) and cells stimulated with either soluble IL-2 or plate-bound HS/IL-2. A fluorescence minus one (FMO) control, containing all antibodies except the antibody against pSTAT5 is shown as well and was used to establish background signal. **c,** Stat5 phosphorylation in WT and $HPSE^{-/-}$ $Foxp3^+$ Treg after stimulation with soluble IL-2. *P* value determined by two-way ANOVA with Sidak's multiple comparison correction. **d,** Representative plots of the percentage of pStat5+ cells among $CD4^+$ $Foxp3^+$ Treg isolated from naïve WT and $HPSE^{-/-}$ spleen tissue. **e,** Percentage of FOXP3+ Treg among CD4+ T cells in the spleens (lefts panel) and inguinal lymph nodes (right panel) of adult (3 to 6-month-old) wt and $HPSE^{-/-}$ mice. Shown are mean + SEM; *P* values determined by two-tailed t-test. **f,** Percentage of Foxp3+ Treg among CD4+ T cells in the spleens (lefts panel) and inguinal lymph nodes (right panel) of adult (3 to 6-month-old) WT and HPSE-/- mice on the 10BiT background (top panels) C57Bl/6 background (lower panels). *P* values determined by two-tailed t-test. **g,** IFN-γ, IL-17 and TNF-α-producing CD4+ T cell subsets in mesenteric lymph nodes of WT and $HPSE^{-/-}$ mice; n = 3 mice. **h,** Gating scheme showing the frequency of $Foxp3^+$ Treg among transferred ($CD90.2^+$) $CD3^+$/$CD4^+$ T cells derived from WT ($CD45.1^+$) and $HPSE^{-/-}$ ($CD45.2^+$) in the spleen of recipient mice. i**,** Quantification of frequencies of $Foxp3^+$ Treg among transferred $CD45.1^+$ WT and $CD45.2^+$ $HPSE^{-/-}$ among Treg in lymphoid tissues of recipient mice. Shown are mean + SEM; ns – non significant, two-way ANOVA with Sidak's multiple comparison correction.

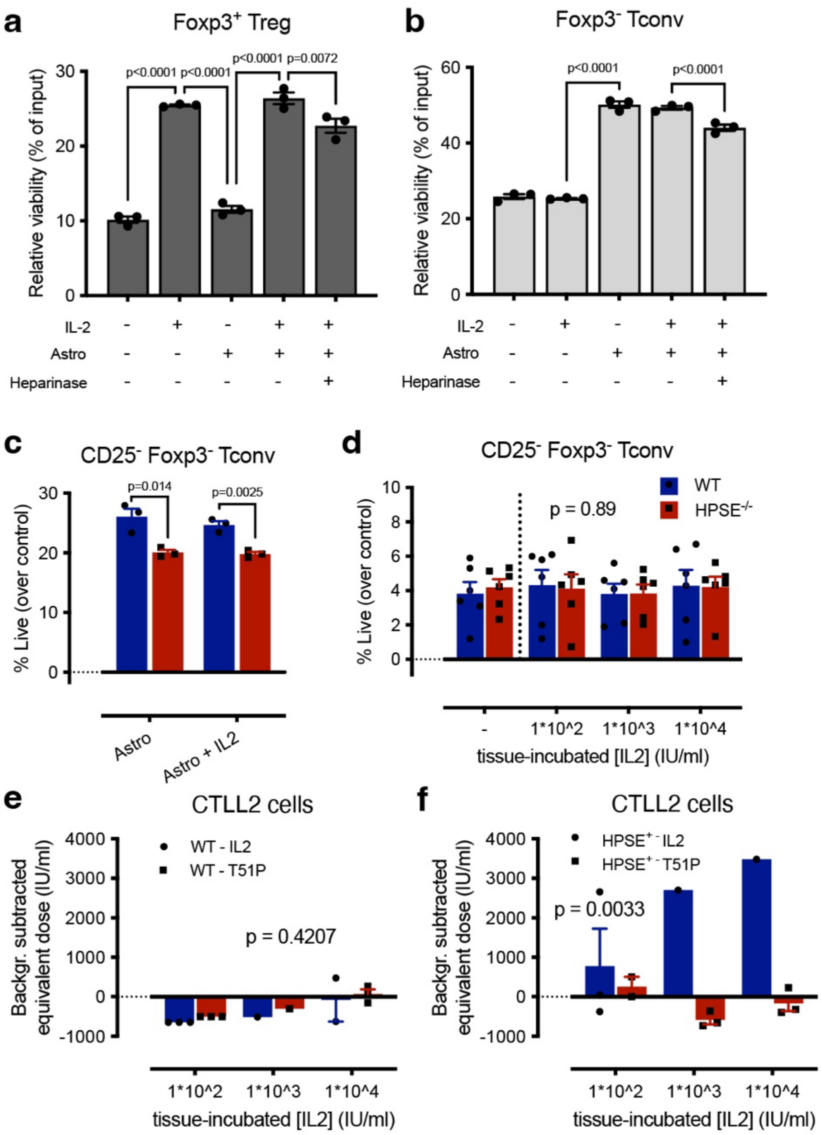

Supplementary Figure 11. HPSE expression supports Foxp3$^+$ Treg access to tissue-bound IL-2 and their function *in vivo*. **a** and **b**, Viability of WT Treg (**a**) and Tconv (**b**) cultured in the presence of U87-MG cells that were pre-incubated with IL-2 alone, or together with heparinase. *P* values determined by two-way ANOVA with Sidak's multiple comparison correction. **c** and **d,** Viability of WT and HPSE$^{-/-}$ CD25$^-$ Foxp3$^-$ Tconv among CD4$^+$ T cells cultured in the presence of U87-MG cells (**c**), or mouse spinal cord tissue (**d**) pre-incubated with IL-2. Viability was measured by flow cytometry 24 hr after start of culture and is shown as percent viable cells corrected for baseline viability of cell cultured in media alone. Shown are representatives of 4 (**c**) or 3 (**d**) independent experiments (mean + SEM of triplicate samples); (**c,** *P* values determined by two-tailed t-test with Holm-Sidak multiple comparison correction; **d,** *P* values depict variation due to the genotype (WT vs. HPSE$^{-/-}$), determined by two-way ANOVA. **e** and **f,** Proliferation of (**e**) WT and (**f**) HPSE overexpressing CTLL2 cells in response to freshly isolated and irradiated mouse spinal cord tissue that was pre-incubated with IL-2 or T51P-IL-2.

Proliferation rate is depicted as equivalent dose of soluble IL2 or T51P-IL-2 at which a similar proliferative response is elicited, corrected for background proliferation induced by spinal cord tissue alone. Shown are mean + SEM of 1-3 wells per sample, *P* value depicts variation due to the cytokine that the beads were incubated with (IL-2 vs. T51P-IL-2), determined by two-way ANOVA.

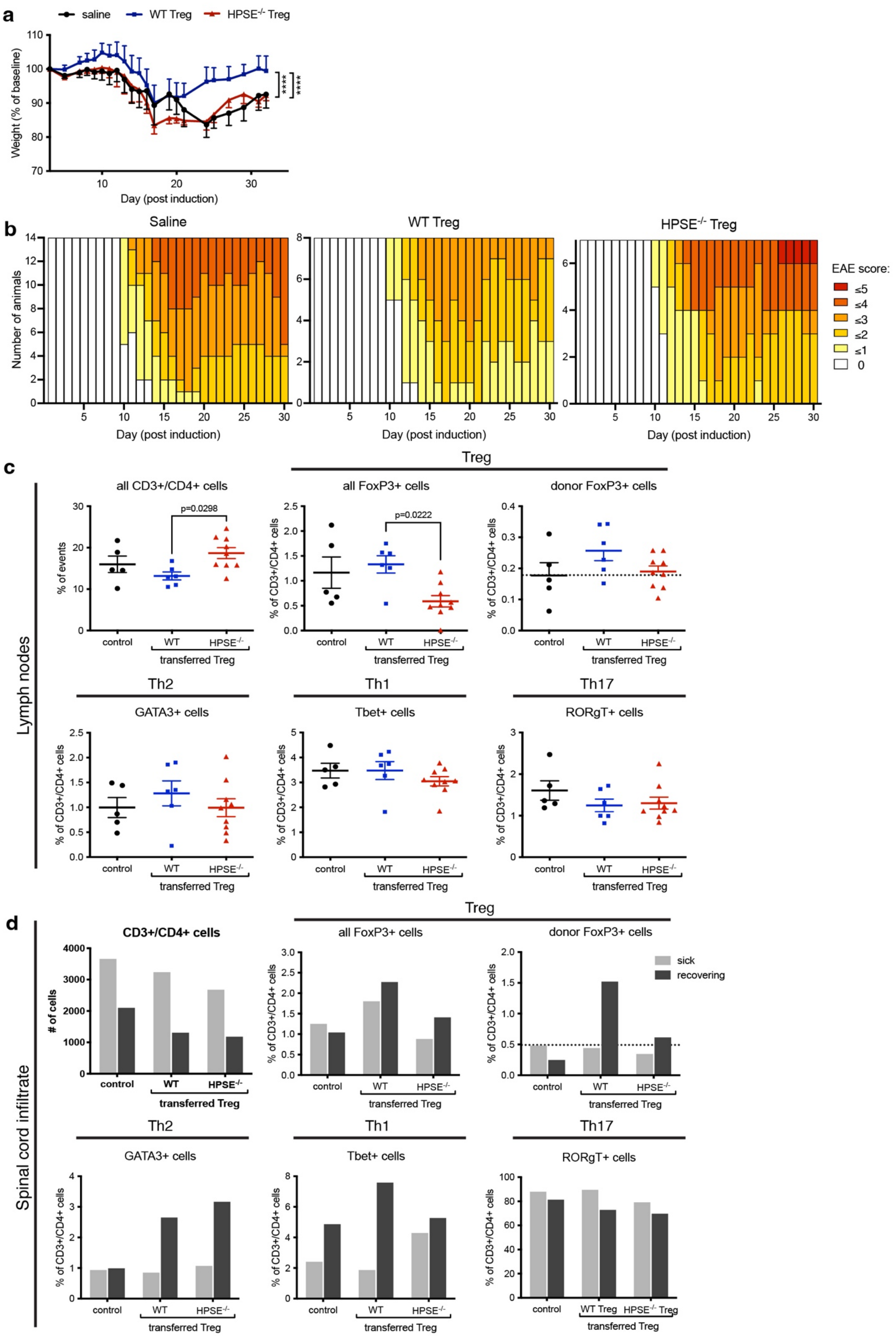
a
saline
WT Treg
HPSE-/- Treg
Weight (% of baseline)
Day (post induction)
****
****
b
Saline
WT Treg
HPSE-/- Treg
Number of animals
Day (post induction)
EAE score:
≤5
≤4
≤3
≤2
≤1
0
c
Lymph nodes
Treg
all CD3+/CD4+ cells
p=0.0298
% of events
all FoxP3+ cells
p=0.0222
% of CD3+/CD4+ cells
donor FoxP3+ cells
control
WT
HPSE-/-
transferred Treg
Th2
GATA3+ cells
Th1
Tbet+ cells
Th17
RORgT+ cells
d
Spinal cord infiltrate
CD3+/CD4+ cells
# of cells
all FoxP3+ cells
donor FoxP3+ cells
sick
recovering
Th2
GATA3+ cells
Th1
Tbet+ cells
Th17
RORgT+ cells
WT Treg
HPSE-/- Treg
transferred Treg

Supplementary Figure 12. HPSE expression supports Treg suppression of EAE. a, Weights of EAE -afflicted animals that received WT or HPSE$^{-/-}$ Foxp3$^+$ Treg 1 day prior to induction of EAE. Average weight +/- SEM are shown as percentage of original weight at d0. *P* values determined by two-way ANOVA with Tukey's multiple comparison correction between WT Treg and control, and WT Treg and HPSE$^{-/-}$ Treg. Shown is a representative of 2 independent experiments. **b,** Distribution of disease scores among the treatment groups. The number of animals with indicated color-coded severity scores are shown. **c,** Frequencies of all CD3$^+$/CD4$^+$ cells, and all and donor-derived Foxp3+ cells, as well as Th2 (Gata3+), Th1 (Tbet) and Th17 (RORgt+) cells among CD3$^+$/CD4$^+$ cells isolated from lymph nodes at d38 after induction of EAE. Shown are percentages in individual mice, as well as mean +/- SEM; *P* values determined by one-way ANOVA with Tukey's multiple comparison correction. **d,** Total numbers of CD3$^+$/CD4$^+$ cells, and frequencies of all and donor-derived Foxp3+ cells, as well as Th2 (Gata3+), Th1 (Tbet) and Th17 (RORgt+) cells among CD3$^+$/CD4$^+$ cells recovered from pooled spinal cords (n=3 for each group) of recipient mice that remained paralyzed (sick) or showed consistent recovery (recovery), at d38 post immunization. Dotted line represents false-positive levels due to background fluorescence.

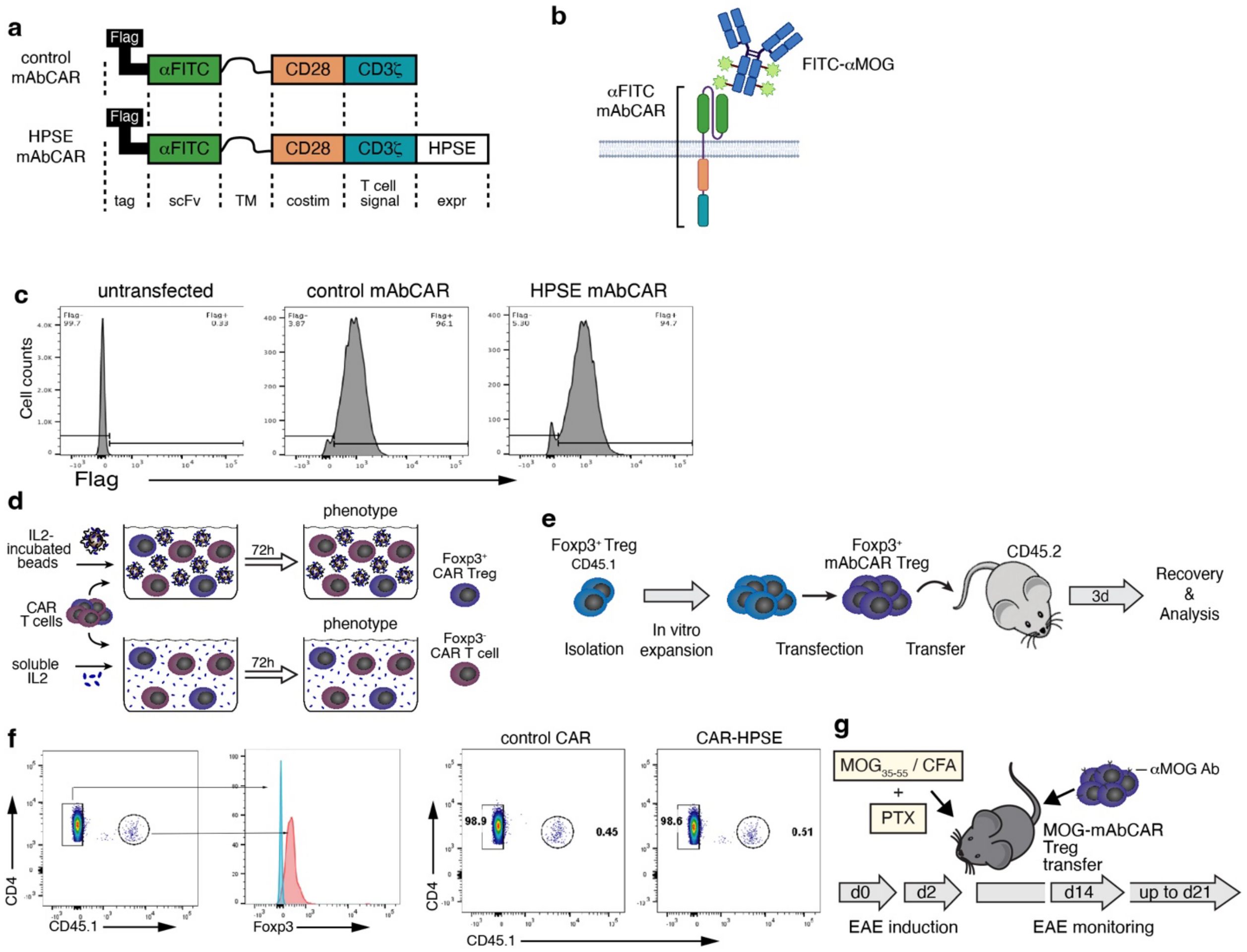

Supplementary Figure 13. HPSE overexpression enhances phenotypic stability of mAbCAR-Treg. a, Schematic and **b,** graphical overview of the monoclonal antibody-chimeric antigen receptor (mAbCAR) constructs used to transfect into Treg. Abbreviations: scFv - single-chain variable region; TM - transmembrane domain; costim - costimulatory signaling domain; T cell signal - T cell receptor signaling domain; expr - expression module. **c,** Flow cytometry histograms depicting the transfection efficiency of the mAbCAR construct used. Efficiency was determined by staining for the FLAG tag present in the constructs. Shown are representative plots of 3 independent experiments. **d,** Schematic overview of the assay used to assess in vitro stability of mAbCAR-Treg (either control, or overexpressing HPSE) induced by heparin-coated beads pre-incubated with IL-2, or soluble IL-2. **e,** Schematic overview of the experiment designed to assess the *in vivo* stability of transferred control and HPSE mAbCAR Treg (Foxp3+). **f,** Identification strategy (left panels) and frequency (right panels) of control and HPSE overexpressing mAbCAR-Tregs (CD45.1+) among CD4+ T cells in lymphoid tissue, 3 days after adoptive transfer of these cells into C57Bl/6 recipients (CD45.2+). **g,** Schematic overview of the experiment designed to assess the capacity of transferred control and HPSE-overexpressing MOG-specific mAbCAR Treg (Foxp3+) to suppress established EAE.

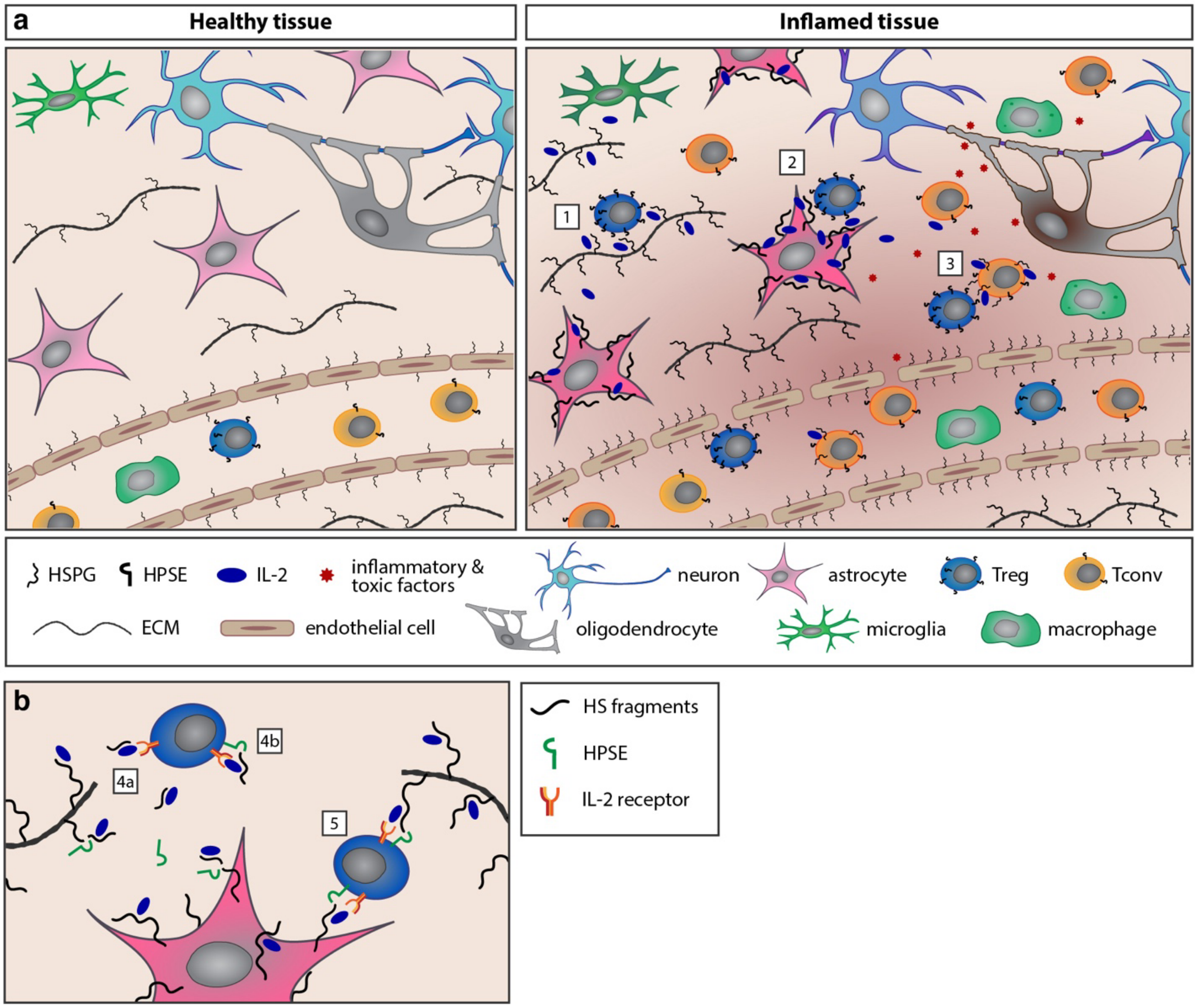

Supplementary Figure 14. Model of Treg access to HS-bound IL-2 in inflamed tissue. a, We propose that HPSE expression enables Treg to access IL-2 that is sequestered to HS. In inflamed tissues, such as MS lesions, activated Treg, which express higher levels of HPSE than activated Tconv, encounter enhanced levels of HS and/or IL-2 in several ways: 1) Treg can access IL-2 that is deposited on HSPGs in the ECM. This IL-2 is likely produced by CNS resident immunocompetent cells (microglia and astrocytes), as well as infiltrating effector T cells. 2) HS and IL-2 are present on reactive astrocytes and HPSE expression enables Treg to access this IL-2. 3) Finally, Treg could encounter IL-2 that is produced by and bound to HS on the cell surface of neighboring activated Tconv. **b,** HPSE could act to enhance the access of Treg to IL-2, either by cleaving and liberating sequestered IL-2 into HS/IL-2 fragments (4a), and/or to facilitate or enhance the interaction of IL-2, bound to HS fragments (4b), or HSPG in the ECM or on cell surfaces (5), with IL-2R complex.

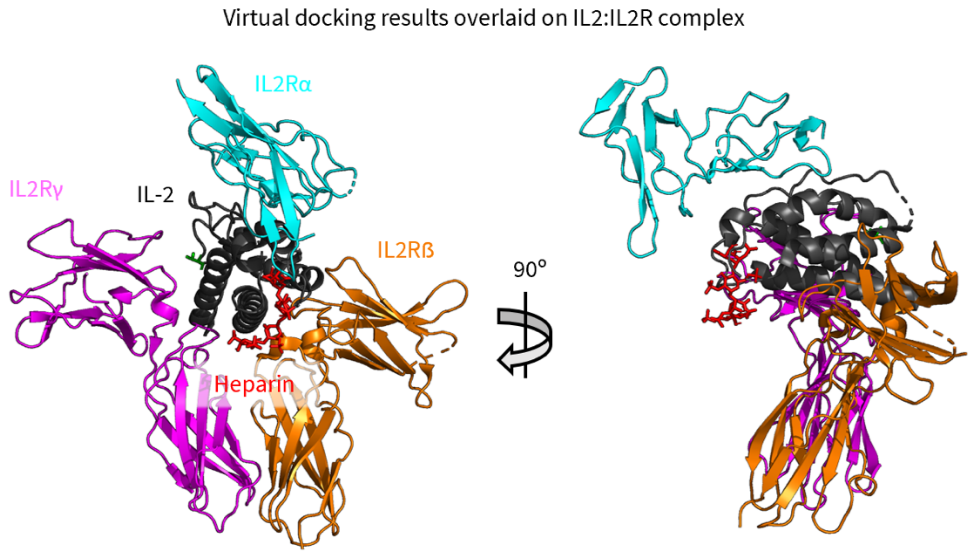

Supplementary Figure 15. Model of the interaction between heparin and IL-2 in the context of the IL-2R complex. Using the ClusPro web server for protein-protein docking, we submitted PDB 2B5I (IL2:IL2R complex) chain A, and docked a heparin tetrasaccharide, using heparin docking mode. We then overlaid the heparin/IL-2 docked structure into the IL2R complex (PDB 2B5I) with heparin tetrasaccharide depicted in red.

Supplementary Table 1.
EAE statistics. Shown is a representative of 2 independent experiments.

Experimental group	Incidence (%)	Disease onset† (day)	Peak severity† (score)	Average severity† d12-30 (score)
**Control**	100% (14/14)	10.8 ± 0.4	3.5 ± 0.2	2.5 ± 0.2
**WT Treg**	100% (8/8)	11.0 ± 0.3	2.6 ± 0.2*	1.7 ± 0.2*
**HPSE$^{-/-}$ Treg**	100% (7/7)	11.0 ± 0.3	3.5 ± 0.3****	2.6 ± 0.4

† Listed are mean values +/- SEM per group
* $p<0.05$, **** $p<0.0001$, Kruskal-Wallis test comparing WT Treg vs. control or HPSE$^{-/-}$ Treg vs. WT Treg.

Supplementary Table 2: Antibody information

HISTOLOGY					
**Antibody**	**Clone**	**Fluoro-phore**	**Supplier Name**	**Catalog Number**	**Dilution**
Goat anti-GFAP	poly	n/a	Sigma-Aldrich	SAB2500462-100UG	1:1000
Chicken anti-IL2	poly	n/a	Sigma	GW22461F	1:200
Mouse anti-HS	F58-10E4	n/a	Seikagaku	370255-1	1:100
Rat anti-CD45	30-F11	n/a	Life Technologies	MCD4500	1:750
Donkey anti-Rat	poly	AF488	Cedarlane Labs	712-547-003	1:400
Donkey anti-Chicken	poly	AF594	Cedarlane Labs	703-585-155	1:400
Donkey anti-Mouse IgM	poly	AF647	Cedarlane Labs	715-606-020	1:400
Donkey anti-Goat	poly	AF488	Cedarlane Labs	705-547-003	1:400
Donkey anti-Goat	poly	AF647	Thermo Scientific	A-21447	1:400
Donkey anti-Rat	poly	AF647	Cedarlane Labs	712-607-003	1:400
Donkey anti-Rat	poly	AF488	Cedarlane Labs	712-547-003	1:400
Donkey anti-Rat	poly	AF594	Jackson ImmunoResearch	712-585-153	1:400

FLOW CYTOMETRY				
**Antibody**	**Fluorophore**	**Clone**	**Supplier Name**	**Dilution**
anti-CD3	BV785	17A2	Biolegend	1:100
anti-CD3	PE-Cy7	17A2	Biolegend	1:100
anti-CD4	BV421	RM4-5	Biolegend	1:100
anti-CD4	BV785	RM4-5	Biolegend	1:100
anti-CD45.1	AF700	A20	Biolegend	1:100
anti-CD45.2	BV421	104	Biolegend	1:100
anti-CD8	BV711	53-6.7	Biolegend	1:100
anti-CD25	APC-Cy7	PC61	Biolegend	1:100
anti-IFNg	APC	XMG1.12	eBioscience	1:50
anti-IL-17	PE	TC11-18H10.1	Biolegend	1:50
anti-TNFa	PE	MP6-XT22	eBioscience	1:50
anti-pSTAT5 (Y694)	PE	47/Stat5(pY694)	BD Biosciences	1:25 (primary)<br>1:50 (cell line)
anti-human CD4	FITC	RPA-T4	Biolegend	1:100
anti-human CD25	PE-Cy7	M-A251	Biolegend	1:100
anti-human CD127	PerCP-Cy5.5	A019D5	Biolegend	1:100

FUNCTIONAL ANTIBODIES			
**Antibody**	**Clone**	**Supplier Name**	**Dilution**
anti-Mouse CD3	145-2C11	Biolegend	2.5 ug/ml
anti-Mouse CD28	37.51	Biolegend	0.5 ug/ml
anti-IL-2	JES6-1	eBioscience	1:5 Ratio (w/w) w/IL-2

SORTING OF HUMAN DONOR DERIVED T CELLS				
**Antibody**	**Fluorophore**	**Clone**	**Supplier Name**	**Dilution**
anti-CD11c	APC	Bu15	eBioscience	1:100
anti-CD4	AmCyan	RPA-T4	BD Biosciences	1:100
anti-CD45RA	FITC	HI100	BD Biosciences	1:100
anti-CD19	PE-TexasRed	HIB19	BD Biosciences	1:100
anti-CD161	PerCP-Cy5.5	HP-3G10	eBioscience	1:100
anti-CD127	Qdot655	A019D5	Biolegend	1:100
anti-CD25	PE	4E3	Miltenyi	1:100

WESTERN BLOT			
**Antibody**	**Clone**	**Supplier Name**	**Dilution**
anti-Mouse HPSE	PAA711Mu04	CloudClone Corp.	1:400
anti-human HPSE	HP3/17	ProSpec	1:1000
anti-Mouse beta actin	Poly6221	Biolegend	1:7500
anti-Mouse beta actin	CAB340Hu	CloudClone Corp	1:7500
anti-Mouse GAPDH	6C5	Invitrogen	1 ug/ml
