# Supplementary material for: Regulatory T cells use heparanase to access IL-2 bound to extracellular matrix in inflamed tissue
Source: Nat Commun. 2024 Feb 20;15:1564. doi: 10.1038/s41467-024-45012-9 (PMC10879116; doi:10.1038/s41467-024-45012-9)
Supplement: Supplementary file 3 — Description of Additional Supplementary Files [file 41467_2024_45012_MOESM3_ESM.pdf]

# <u>Description of Additional Supplementary Files</u>

**Supplementary Movie Legend**:

**Supplementary Movie 1**. HS and IL2 colocalize in inflamed CNS tissue. 3D rendering of a z2 stack of EAE spinal cord tissue (29 dpi) stained for IL-2 (green), HS (magenta), CD45 (yellow) 3 and DAPI (blue).
