# Supplementary material for: Regulatory T cells use heparanase to access IL-2 bound to extracellular matrix in inflamed tissue
Source: Nat Commun. 2024 Feb 20;15:1564. doi: 10.1038/s41467-024-45012-9 (PMC10879116; doi:10.1038/s41467-024-45012-9)
Supplement: Supplementary file 5 — Reporting Summary [file 41467_2024_45012_MOESM5_ESM.pdf]

nature portfolio

Corresponding author(s): Hedwich Kuipers, Paul Bollyky

Last updated by author(s): Oct 30, 2023

Reporting Summary

Nature Portfolio wishes to improve the reproducibility of the work that we publish. This form provides structure for consistency and transparency in reporting. For further information on Nature Portfolio policies, see our Editorial Policies and the Editorial Policy Checklist.

Statistics

For all statistical analyses, confirm that the following items are present in the figure legend, table legend, main text, or Methods section.

n/a	Confirmed	
☐	☒	The exact sample size (*n*) for each experimental group/condition, given as a discrete number and unit of measurement
☐	☒	A statement on whether measurements were taken from distinct samples or whether the same sample was measured repeatedly
☐	☒	The statistical test(s) used AND whether they are one- or two-sided<br>*Only common tests should be described solely by name; describe more complex techniques in the Methods section.*
☒	☐	A description of all covariates tested
☒	☐	A description of any assumptions or corrections, such as tests of normality and adjustment for multiple comparisons
☐	☒	A full description of the statistical parameters including central tendency (e.g. means) or other basic estimates (e.g. regression coefficient) AND variation (e.g. standard deviation) or associated estimates of uncertainty (e.g. confidence intervals)
☐	☒	For null hypothesis testing, the test statistic (e.g. *F*, *t*, *r*) with confidence intervals, effect sizes, degrees of freedom and *P* value noted<br>*Give P values as exact values whenever suitable.*
☒	☐	For Bayesian analysis, information on the choice of priors and Markov chain Monte Carlo settings
☒	☐	For hierarchical and complex designs, identification of the appropriate level for tests and full reporting of outcomes
☒	☐	Estimates of effect sizes (e.g. Cohen's *d*, Pearson's *r*), indicating how they were calculated

Our web collection on statistics for biologists contains articles on many of the points above.

Software and code

Policy information about availability of computer code

Data collection	No software was used for data collection.
Data analysis	For image analysis Imaris imaging software (Bitplane) was used. For flow cytometry analysis, FlowJo version 9 or 10 (Treestar) was used. For graphing and statistical analysis GraphPad Prism 8.4.2 was used.

For manuscripts utilizing custom algorithms or software that are central to the research but not yet described in published literature, software must be made available to editors and reviewers. We strongly encourage code deposition in a community repository (e.g. GitHub). See the Nature Portfolio guidelines for submitting code & software for further information.

Data

Policy information about availability of data

All manuscripts must include a data availability statement. This statement should provide the following information, where applicable:

- Accession codes, unique identifiers, or web links for publicly available datasets
- A description of any restrictions on data availability
- For clinical datasets or third party data, please ensure that the statement adheres to our policy

Data sharing is not applicable to this article as no datasets were generated or analyzed during the current study.

Research involving human participants, their data, or biological material

Policy information about studies with human participants or human data. See also policy information about sex, gender (identity/presentation), and sexual orientation and race, ethnicity and racism.

Reporting on sex and gender	Sex and gender were not considered in the study from which data is used in this manuscript. Sex, but not gender, was reported, but data was not disaggregated for this. Due to the nature of the disorder of interest (ulcerative colitis and Crohn's disease), the majority of the participants were female (67%), and the control group was matched for this.
Reporting on race, ethnicity, or other socially relevant groupings	No data on race or ethnicity was collected in this study.
Population characteristics	Mean age: 41.4 years (patients), 45 years (controls)<br>% male: 33% (patients), 33% (controls)<br>Mean disease duration (patients): 7.8 years<br>% on steroid treatment (patients): 53%
Recruitment	Individuals 16 years of age and older with known or suspected history of Crohn's disease, Ulcerative Colitis were identified in various locally available databases. Study staff generated a patient list from Cerner to alert them to up-coming clinic visits by potential study candidates. Study staff then contacted clinical staff to ask patients if they would consider participation in a study. Agreeable patients were then formally interviewed and consented. In addition, mailers were sent to patients on the list, inviting them to reply if they would like to participate in IBD research at the BRI. Responders were be invited to the Clinical Research Center (CRC) or another appropriate location convenient to the participant for interview, consent and initial phlebotomy. For control samples, family members and friends were directly referred from probands, and their intake occurred at the CRC or at another location convenient to the participant. There is the potential for self-selection bias amongst our controls given we enrolled from probands rather than random age-matched, sex-matched, healthy controls. This enrollment strategy is likely to impact the results by obfuscating differences driven by the diseases studied.
Ethics oversight	Approval for blood and colon tissue collection was obtained through the Benaroya Research Institute Institutional Review Board (No. 10090).

Note that full information on the approval of the study protocol must also be provided in the manuscript.

Field-specific reporting

Please select the one below that is the best fit for your research. If you are not sure, read the appropriate sections before making your selection.

☒ Life sciences ☐ Behavioural & social sciences ☐ Ecological, evolutionary & environmental sciences

For a reference copy of the document with all sections, see nature.com/documents/nr-reporting-summary-flat.pdf

Life sciences study design

All studies must disclose on these points even when the disclosure is negative.

Sample size	No sample-size calculation was performed. Sample sizes were determined on previous experience with similar experiments yielding statistically significant differences between groups, using appropriate tests. Specifically we used other references (PMID: 33505023; PMID: 23436224) to base our in vivo EAE mouse numbers and replicate number of experiments.
Data exclusions	No data were excluded from any analysis
Replication	In vivo experiments were replicated in at least 2 separate experiments (adoptive transfer studies), or at least 3 separate cohorts/litters of mice (phenotyping studies). In vitro experiments were replicated at least 3 times, with different preparations of cells. Positive and negative controls were included to determine the validity of each replicate experiment. Attempts at replication that did not replicate the findings were only excluded based on these controls.
Randomization	Recipient mice in adoptive transfer experiments randomly received donor T cell suspensions by alternating littermates. For in vitro cell assays randomization was not done. Cells from different backgrounds (e.g. WT vs HPSE-/-) were always plated across equivalent conditions to isolate effects coming from the cells background. Covariates, such as position in the assay plate, were controlled during in vitro assays by having technical replicates within the same plate and did not see a position effect under the conditions tested.
Blinding	For phenotyping/homeostasis analysis of wt vs HPSEko T cells, investigators were blinded, as genotyping results were only available after data collection and analysis. For EAE experiments, some investigators that scored disease severity were blinded. For other experiments, investigators were not blinded. The other experiments were not blinded because they were setup by the same experimentalist designing the assay, executing the assay, and analyzing the results. Blinding was not technically feasible as it would require an additional experimentalist during the execution of the assay.

Reporting for specific materials, systems and methods

We require information from authors about some types of materials, experimental systems and methods used in many studies. Here, indicate whether each material, system or method listed is relevant to your study. If you are not sure if a list item applies to your research, read the appropriate section before selecting a response.

Materials & experimental systems

n/a	Involved in the study	
☐	☒	Antibodies
☐	☒	Eukaryotic cell lines
☒	☐	Palaeontology and archaeology
☐	☒	Animals and other organisms
☒	☐	Clinical data
☒	☐	Dual use research of concern
☒	☐	Plants

Methods

n/a	Involved in the study	
☒	☐	ChIP-seq
☐	☒	Flow cytometry
☒	☐	MRI-based neuroimaging

Antibodies

Antibodies used

HISTOLOGY
Antibody Clone Fluorophore Supplier Name Catalog Number Dilution
Goat anti-GFAP poly n/a Sigma-Aldrich SAB2500462-100UG 1:1000
Chicken anti-IL2 poly n/a Sigma GW22461F 1:200
Mouse anti-HS F58-10E4 n/a Seikagaku 370255-1 1:100
Rat anti-CD45 30-F11 n/a Life Technologies MCD4500 1:750
Donkey anti-Rat - AF488 poly AF488 Cedarlane Labs 712-547-003 1:400
Donkey anti-Chicken - AF594 poly AF594 Cedarlane Labs 703-585-155 1:400
Donkey anti-Mouse IgM - AF647 poly AF647 Cedarlane Labs 715-606-020 1:400
Donkey anti-Goat AF488 poly AF488 Cedarlane Labs 705-547-003 1:400
Donkey anti-Goat AF647 poly AF647 Thermo Scientific A-21447 1:400
Donkey anti-Rat AF647 poly AF647 Cedarlane Labs 712-607-003 1:400
Donkey anti-Rat AF488 poly AF488 Cedarlane Labs 712-547-003 1:400
Donkey anti-Rat AF594 poly AF594 Jackson ImmunoResearch 712-585-153 1:400

FLOW CYTOMETRY
Antibody, Fluorophore, Clone, Supplier Name, Dilution
anti-CD3, BV785, 17A2, Biolegend, 1:100
anti-CD3, PE-Cy7, 17A2, Biolegend, 1:100
anti-CD4, BV421, RM4-5, Biolegend, 1:100
anti-CD4, BV785, RM4-5, Biolegend, 1:100
anti-CD45.1 AF700 A20 Biolegend 1:100
anti-CD45.2 BV421 104 Biolegend 1:100
anti-CD8, BV711, 53-6.7, Biolegend, 1:100
anti-CD25, APC-Cy7, PC61, Biolegend, 1:100
anti-IFNg, APC, XMG1.12, eBioscience, 1:50,
anti-IL-17, PE,, TC11-18H10.1, Biolegend, 1:50,
anti-TNFa, PE, MP6-XT22, eBioscience, 1:50,
anti-pSTAT5 (Y694), PE, 47/Stat5(pY694), BD Biosciences, 1:25 (primary), 1:50 (cell line)
anti-human CD4, FITC, RPA-T4, Biolegend, 1:100
anti-human CD25, PE-Cy7, M-A251, Biolegend, 1:100
anti-human CD127, PerCP-Cy5.5, A019D5, Biolegend, 1:100

FUNCTIONAL ANTIBODIES
Antibody, Clone, Supplier Name, Dilution
anti-mouse CD3, 145-2C11, Biolegend, 2.5 ug/ml
anti-mouse CD28, 37.51, Biolegend, 0.5 ug/ml
anti-IL-2, JES6-1, eBioscience, 1:5 ratio (w/w) w/IL-2

SORTING OF HUMAN DONOR DERIVED T CELLS
Antibody, Fluorophore, Clone, Supplier Name, Dilution
anti-CD11c, APC, Bu15, eBioscience, 1:100
anti-CD4, AmCyan, RPA-T4, BD Biosciences, 1:100
anti-CD45RA, FITC, HI100, BD Biosciences, 1:100
anti-CD19, PE-TexasRed, HIB19, BD Biosciences, 1:100
anti-CD161, PerCP-Cy5.5, HP-3G10, eBioscience, 1:100
anti-CD127, Qdot655, A019D5, Biolegend, 1:100
anti-CD25, PE, 4E3, Miltenyi, 1:100

WESTERN BLOT

Antibody, Clone, Supplier Name, Dilution
anti-mouse HPSE, PAA711Mu04, CloudClone Corp., 1:400
anti-human HPSE, HP3/17, ProSpec, 1:1000
anti-mouse beta actin, Poly6221, Biolegend, 1:7500
anti-mouse beta actin, CAB340Hu, CloudClone Corp., 1:7500
anti-mouse GAPDH, 6C5, Invitrogen, 1 ug/ml

Validation

Validation data on the manufacturer's website was verified for all antibodies used. In addition, specificity of the chicken anti-IL2 antibody (detects rat, mouse and human IL-2) was determined using IL2-/- tissue (histology) and tissue lysates (Western Blot).
HISTOLOGY
mouse anti-HS, n/a F58-10E4, Seikagaku. Validated by manufacturer using heparinase to abolish staining and by citation (PubMed ID: 36494335).
rat anti-CD45, clone 30-F11. Validated by manufacturer using isotype and by citation (PMID: 33659003). RRID: AB_312966.
donkey anti-rat - AF488, poly, Cedarlane Labs, 712-547-003, 1:1000. No validation available.
donkey anti-chicken - AF594, poly, Cedarlane Labs, 703-585-155, 1:1000. No validation available.
donkey anti-mouse IgM - AF647 poly, Cedarlane Labs, 715-606-020, 1:1000. No validation available.

FLOW CYTOMETRY
Antibody, Fluorophore, Clone, Supplier Name
anti-CD3, BV785, 17A2, Biolegend. Validated by manufacturer using isotype and by citation (PMID: 33376221). RRID: AB_11218805.
anti-CD3, PE-Cy7, 17A2, Biolegend. Validated by manufacturer using isotype and by citation (PMID: 33376221). RRID: AB_1732068.
anti-CD4, BV421, RM4-5, Biolegend. Validated by manufacturer using isotype and by citation (PMID: 17158224). RRID: AB_10898318.
anti-CD4, BV785, RM4-5, Biolegend. Validated by manufacturer using isotype and by citation (PMID: 17158224). RRID: AB_11218992.
anti-CD45.1, AF700, A20, Biolegend. Validated by manufacturer. (PMID: 25135834). RRID: AB_493732
anti-CD45.2, BV421,104, Biolegend. Validated by manufacturer. (PMID: 30995471). RRID: AB_10900256
anti-CD8, BV711, 53-6.7, Biolegend. Validated by manufacturer using isotype and by citation (PMID: 34289354). RRID: AB_11219594.
anti-CD25, APC-Cy7, PC61, Biolegend. Validated by manufacturer using isotype and by citation (PMID: 31079916). RRID: AB_830744.
anti-IFNg, APC, XMG1.12, eBioscience. Validated by manufacturer using isotype and by citation (PMID: 34294718). RRID: AB_315403.
anti-IL-17, PE, TC11-18H10.1, Biolegend. Validated by manufacturer using isotype and by citation (PMID: 19129757). RRID: AB_315463.
anti-TNFa, PE, MP6-XT22, eBioscience. Validated by manufacturer using isotype and by citation (PMID: 35915084). RRID:AB_315426.
anti-pSTAT5 (Y694), PE, 47/Stat5(pY694), BD Biosciences. Validated by manufacturer using isotype. RRID:AB_399858.
anti-human CD4, FITC, RPA-T4, Biolegend. Validated by manufacturer using isotype and by citation (PMID: 19773555). RRID: AB_314073.
anti-human CD25, PE-Cy7, M-A251, Biolegend. Validated by manufacturer using isotype and by citation (PMID: 35022622). RRID:AB_2561860.
anti-human CD127, PerCP-Cy5.5, A019D5, Biolegend. Validated by manufacturer using isotype and by citation (PMID: 31027998). RRID: AB_10900253.

FUNCTIONAL ANTIBODIES
Antibody, Clone, Supplier Name
anti-mouse CD3, 145-2C11, Biolegend. Validated by manufacturer and by citation (PMID: 31324723). RRID: AB_312666.
anti-mouse CD28, 37.51, Biolegend. Validated by manufacturer and by citation (PMID: 26840450). RRID: AB_312866.
anti-IL-2, JES6-1, eBioscience. Validated by manufacturer and by citation (PMID: PMID: 27141363). RRID: AB_469206

SORTING OF HUMAN DONOR DERIVED T CELLS
Antibody, Fluorophore, Clone, Supplier Name
anti-CD11c, APC, Bu15, eBioscience. Validated by manufacturer using isotype. RRID: AB_11151141.
anti-CD4, AmCyan, RPA-T4, BD Biosciences. Validated by manufacturer using isotype.
anti-CD45RA, FITC, HI100, BD Biosciences. Validated by manufacturer using isotype.
anti-CD19, PE-TexasRed, HIB19, BD Biosciences. Validated by manufacturer using isotype. RRID: AB_395813.
anti-CD161, PerCP-Cy5.5, HP-3G10, eBioscience. Validated by manufacturer using isotype and by citation (PMID: 35829840). RRID: AB_1311148.
anti-CD127, Qdot655, A019D5, Biolegend. Validated by manufacturer using isotype and by citation (PMID: 31209406). RRID: AB_2562019.
anti-CD25, PE, 4E3, Miltenyi. Validated by manufacturer using isotype and compared against known hybridomas. RRID: AB_2734062.

WESTERN BLOT
Antibody, Clone, Supplier Name
anti-mouse HPSE, PAA711Mu04, CloudClone Corp. Validated by citation (PMID: 26191183).
anti-human HPSE, HP3/17, ProSpec. Validated by citation (PMID: 11406531).
anti-mouse beta actin, Poly6221, Biolegend. Validated by citation (PMID: 32232903). RRID: AB_315945
anti-mouse beta actin, CAB340Hu CloudClone Corp., Validated by manufacturer.
anti-mouse GAPDH, 6C5, Invitrogen. Validate by citation (PMID: 36763621). RRID: AB_2536381
Validation data on the manufacturer's website was verified for all antibodies used. In addition, specificity of the chicken anti-IL2 antibody (detects rat, mouse and human IL-2) was determined using IL2-/- tissue (histology) and tissue lysates (Western Blot).
HISTOLOGY
mouse anti-HS, n/a F58-10E4, Seikagaku, 1:1000. Validated by manufacturer using heparinase to abolish staining and by citation (PubMed ID: 36494335).
rat anti-CD45, clone 30-F11. Validated by manufacturer using isotype and by citation (PMID: 33659003). RRID: AB_312966.
donkey anti-rat - AF488, poly, Cedarlane Labs, 712-547-003, 1:1000. No validation available.

donkey anti-chicken - AF594, poly, Cedarlane Labs, 703-585-155, 1:1000. No validation available.
donkey anti-mouse IgM - AF647 poly, Cedarlane Labs, 715-606-020, 1:1000. No validation available.

Eukaryotic cell lines

Policy information about cell lines and Sex and Gender in Research

Cell line source(s)	CTLL-2 cells were acquired from the American Type Culture Collection (ATCC). These cells are derived from a cytotoxic T cell from a C57BL/6 mouse.
Authentication	CTLL-2 cells were authenticated by verifying dependence on IL-2 for growth and survival. No other means of authentication were measured.
Mycoplasma contamination	Cell lines were not tested for mycoplasma contamination.
Commonly misidentified lines (See ICLAC register)	CTLL-2 (CVCL_0227) was used in this study. The rationale for its use in this study is it requires IL-2 for its growth and survival, thus making it useful tool to study IL-2 signaling biology. No commonly misidentified lines were used in this study.

Animals and other research organisms

Policy information about studies involving animals; ARRIVE guidelines recommended for reporting animal research, and Sex and Gender in Research

Laboratory animals	To generate HPSE-/- -FOXP3.GFP mice, harboring a GFP-FOXP3 fusion reporter knock-in allele in addition to a targeted disruption of the HPSE gene, HPSE-/- mice, described before1 and kind gift of Drs. Israel Vlodavsky and Jin-Ping Li, were crossed with FOXP3.GFP reporter mice2, a kind gift of Dr. Alexander Rudensky. Both strains were maintained on the C57BL/6J background and offspring was backcrossed and maintained as a homozygous line. In addition, resulting HPSE-/- -FOXP3.GFP mice were crossed with 10BiTFOXP3. GFP mice, derived from crossing 10BiT mice containing a Thy1.1 (CD90.1) reporter under the control of the IL-10 promoter and FOXP3.GFP reporter mice3, a kind gift of Dr. Casey Weaver. Wild-type (CD45.2) and congenic CD45.1 C57BL/6J, C.129P2(B6)-Il2tm1Hor/J (IL2-/-) and B6.129S7-Rag1tm1Mom/J (RAG1-/-) mice were obtained from Jackson Laboratories (Bar Harbor, ME). Congenic CD90.1 C57BL/6J were a kind gift of Dr. Robert Negrin.<br>1. Zcharia, E. et al. Newly Generated Heparanase Knock-Out Mice Unravel Co-Regulation of Heparanase and Matrix Metalloproteinases. PLoS One 4, e5181 (2009).<br>2. Fontenot, J. D. et al. Regulatory T Cell Lineage Specification by the Forkhead Transcription Factor Foxp3. Immunity 22, 329–341 (2005).<br>3. Maynard, C. L. et al. Regulatory T cells expressing interleukin 10 develop from Foxp3+ and Foxp3- precursor cells in the absence of interleukin 10. Nat. Immunol. 8, 931–941 (2007).<br>Mice were used at 8-12 weeks of age, unless otherwise indicated in the figure legends.
Wild animals	The study did not involve wild animals
Reporting on sex	Male animals were used for experiments, unless indicated otherwise. A comprehensive comparison with female mice was not performed.
Field-collected samples	The study did not involve samples collected from the field.
Ethics oversight	All mice were maintained in specific pathogen-free AAALAC-accredited animal facilities at the BRI and Stanford University and handled in accordance with institutional guidelines.

Note that full information on the approval of the study protocol must also be provided in the manuscript.

Plants

Seed stocks	*Report on the source of all seed stocks or other plant material used. If applicable, state the seed stock centre and catalogue number. If plant specimens were collected from the field, describe the collection location, date and sampling procedures.*
Novel plant genotypes	*Describe the methods by which all novel plant genotypes were produced. This includes those generated by transgenic approaches, gene editing, chemical/radiation-based mutagenesis and hybridization. For transgenic lines, describe the transformation method, the number of independent lines analyzed and the generation upon which experiments were performed. For gene-edited lines, describe the editor used, the endogenous sequence targeted for editing, the targeting guide RNA sequence (if applicable) and how the editor was applied.*
Authentication	*Describe any authentication procedures for each seed stock used or novel genotype generated. Describe any experiments used to assess the effect of a mutation and, where applicable, how potential secondary effects (e.g. second site T-DNA insertions, mosiacism, off-target gene editing) were examined.*

Flow Cytometry

Plots

Confirm that:

☒ The axis labels state the marker and fluorochrome used (e.g. CD4-FITC).

☒ The axis scales are clearly visible. Include numbers along axes only for bottom left plot of group (a 'group' is an analysis of identical markers).

☒ All plots are contour plots with outliers or pseudocolor plots.

☒ A numerical value for number of cells or percentage (with statistics) is provided.

Methodology

Sample preparation	Spleen/lymph node/thymus: tissue was homogenized through a 70 um strainer into RPMI.<br>Bone marrow: femurs were flushed with RPMI.<br>Lungs and large intestine: tissues were minced and digested with 100 IU/ml Collagenase I and 25 IU/ml DNase I (30-45 minutes 37C).<br>Spinal cord: tissue was homogenized using a dounce homogenizer. Cells were then isolated by density centrifugation using a 28% Percoll solution, layered with PBS.<br>Red blood cells were lysed in all suspensions in red blood cell lysis buffer (Sigma).
Instrument	LSRII (Becton Dickinson)
Software	FlowJo Version 9 or 10 (Treestar)
Cell population abundance	CD4+ bead sorted cells (used for all culture experiments and various adoptive transfer experiments), purity was routinely >95%, as determined by flow cytometry.
Gating strategy	Mouse Treg/Tconv (from tissue, or after culture): FSC/SSC Lymphocyte gate > FSC-H/FSC-A single cell gate > SSC-H/SSC-A single cell gate > CD3-BV785/CD4-BV421 CD3+/CD4+ gate > CD25-APC-Cy7/GFP(Foxp3) CD25high/GFP+ gate: Treg; CD25neg-low/GFP- gate: Tconv<br><br>Sorted human T cells from blood : FSC/SSC Lymphocyte gate > FSC-H/FSC-A single cell gate > SSC-H/SSC-A single cell gate > CD4-FITC/FSC-H CD4+ gate > CD25-PE/Cy7/CD127-PerCp-Cy5.5 CD25high/CD127neg: Treg; CD25low/CD127pos gate: Tconv.<br><br>Sorted human T cells from blood and colon samples: gating scheme is provided in Supplementary Information.

☒ Tick this box to confirm that a figure exemplifying the gating strategy is provided in the Supplementary Information.
